# Longitudinal effects of aging on ^18^F-FDG distribution in cognitively normal elderly individuals

**DOI:** 10.1038/s41598-018-29937-y

**Published:** 2018-08-01

**Authors:** Kenji Ishibashi, Airin Onishi, Yoshinori Fujiwara, Keiichi Oda, Kiichi Ishiwata, Kenji Ishii

**Affiliations:** 1 Research Team for Neuroimaging, Tokyo Metropolitan Institute of Gerontology, Tokyo, Japan; 2 Research Team for Social Participation and Community Health, Tokyo Metropolitan Institute of Gerontology, Tokyo, Japan; 3 Faculty of Health Science, Hokkaido University of Science, Sapporo, Japan; 4 Institute of Cyclotron and Drug Discovery Research, Southern Tohoku Research Institute for Neuroscience, Koriyama, Japan; 5 Department of Biofunctional Imaging, Fukushima Medical University, Fukushima, Japan

## Abstract

Previous studies of aging effects on fluorine-18-labeled fluorodeoxyglucose (^18^F-FDG) distribution have employed cross-sectional designs. We examined aging effects on ^18^F-FDG distribution using both cross-sectional and longitudinal assessments. We obtained two ^18^F-FDG positron emission tomography scans at two different time points from 107 cognitively normal elderly participants. The participants’ mean ages at baseline and second scans were 67.9 and 75.7, respectively. The follow-up period ranged from 4 to 11 years with a mean of 7.8 years. The voxel-wise analysis revealed significant clusters in which ^18^F-FDG uptake was decreased between baseline and second scans (*p* < 0.05, family-wise error corrected) in the anterior cingulate cortex (ACC), posterior cingulate cortex/precuneus (PCC/PC), and lateral parietal cortex (LPC). The cross-sectional analysis of ^18^F-FDG uptake and age showed significant correlations in the ACC (*p* = 0.016) but not the PCC/PC (*p* = 0.240) at baseline, and in the ACC (*p* = 0.004) and PCC/PC (*p* = 0.002) at the second scan. The results of longitudinal assessments suggested that ^18^F-FDG uptake in the ACC, PCC/PC, and LPC decreased with advancing age in cognitively normal elderly individuals, and those of the cross-sectional assessments suggested that the trajectories of age-associated ^18^F-FDG decreases differed between the ACC and PCC/PC.

## Introduction

Fluorine-18-labeled fluorodeoxyglucose (^18^F-FDG) is a radioligand used in positron emission tomography (PET) to estimate regional glucose metabolism, which reflects regional brain activity^1^. Although brain activity decreases with advancing age, the magnitude of the reduction differs depending on the brain region^2^. This decreased brain activity leads to cognitive decline in elderly individuals compared to young individuals^3,4^. As the numbers of elderly individuals in the world population and patients with senile dementia are increasing, understanding the effects of aging on brain activity is essential for comprehending the progress of senile dementia, such as Alzheimer’s disease (AD), dementia with Lewy bodies, and frontotemporal dementia.

Since the 1980s, cross-sectional studies have used ^18^F-FDG PET to investigate the association between brain activity and aging effects in young to elderly individuals^5–10^. Although the regions showing the greatest aging effects differed slightly among these studies due to differences in their methodology and sample characteristics^11^, the results of these studies support the so-called frontal aging hypothesis that the anterior regions of the brain are more vulnerable to aging^4^. In particular, recent cross-sectional ^18^F-FDG PET studies have shown that the medial part of the prefrontal cortex, the anterior cingulate cortex (ACC), exhibits highly significant age-associated decreases in ^18^F-FDG uptake^5,6,8,9,12^.

One major drawback of the above cross-sectional studies is that inter-individual variabilities in physiological and anatomical factors at certain ages, such as ^18^F-FDG distribution and brain structure, may affect the findings of aging effects on ^18^F-FDG distribution. Therefore, longitudinal data are essential to better comprehend these effects. Recently, a few studies have addressed the longitudinal changes in ^18^F-FDG distribution^13–15^; however, their focus was on the progression of AD, not aging effects. These studies failed to observe significant effects of aging on ^18^F-FDG distribution in elderly individuals, likely due to smaller sample sizes and shorter follow-up periods^14,15^. The primary aim of this study is to expand the current knowledge of aging effects on ^18^F-FDG distribution by performing longitudinal assessments with a larger number of subjects and a longer follow-up period. To this end, we performed two ^18^F-FDG PET scans on 107 cognitively normal elderly participants with an inter-scan interval ranging from 4 to 11 years and a mean of 7.8 years. We also performed cross-sectional assessments of aging effects on ^18^F-FDG distribution to corroborate longitudinal assessments.

## Materials and Methods

### Research Participants

This prospective study was conducted in accordance with the Helsinki Protocol and approved by the Ethics Committee of the Tokyo Metropolitan Institute of Gerontology. Written informed consent was obtained from all participants. The participants comprised 107 cognitively normal individuals (19 men and 88 women) who were recruited from ongoing longitudinal studies of cognition and aging at the Tokyo Metropolitan Institute of Gerontology^16^. Participants underwent two ^18^F-FDG PET scans with an inter-scan interval of at least 4 years. The mean age at the first and second ^18^F-FDG PET scans were 67.9 ± 4.9 years and 75.7 ± 4.8 years, respectively. The interval between ^18^F-FDG PET scans ranged from 4 to 11 years, with a mean of 7.8 years. Prior to each scan, all participants underwent interviews and clinical examinations by a physician to ensure that all participants were cognitively normal and living independently^17^. The mean Mini-Mental State Examination (MMSE) score ranged from 25 to 30, with a mean of 29.2 ± 1.1 at the second ^18^F-FDG PET scan. Any individuals determined to have a history of diabetes or a neurological, mental health, or other uncontrolled health condition in the physical and neurological examinations and routine mental health interviews were excluded. All participants underwent conventional magnetic resonance imaging (MRI) at the time of the ^18^F-FDG PET scans, and no participants showed significant brain atrophy or lesions in the MRI findings. The handedness of the participants was determined using the Edinburgh Handedness Inventory^18^. Of the 107 participants, 98 and 4 participants were right- and left-handed, respectively. The remaining 5 participants were unknown. Additionally, the apolipoprotein E (ApoE) genotype of each individual was determined. Because individuals with the ApoE ε4 genotype have a higher risk of developing AD^19,20^, we created 3 groups to assess the influence of the ApoE ε4 genotype in addition to the effects of aging on ^18^F-FDG distribution. Group 1 included all participants (n = 107), group 2 consisted of participants without the ApoE ε4 genotype (n = 89), and group 3 consisted of participants with the ApoE ε4 genotype (n = 18). Thus, group 1 was divided into groups 2 and 3. The characteristics of the study participants are summarized in Table 1.

**Table 1:** Characteristics of the study participants.

	Group 1	Group 2*	Group 3*
	n = 107	n = 89	n = 18
Age at the baseline PET (years)	67.9 ± 4.9	67.6 ± 5.1	69.3 ± 3.9
Range	56–81	56–81	62–76
Age at the second PET (years)	75.7 ± 4.8	75.4 ± 4.9	76.8 ± 3.9
Range	64–90	64–90	70–83
MMSE at the second PET	29.2 ± 1.1	29.2 ± 1.0	29.2 ± 1.3
	25–30	26–30	25–30
Interval (years)	7.8 ± 1.8	7.8 ± 1.8	7.6 ± 1.9
Sex			
Male	19	14	5
Female	88	75	13
Apolipoprotein E ε4			
Presence	18	0	18
Absence	89	89	0

The data represent the mean ± standard deviation.

*Group 1 was divided into groups 2 and 3.

MMSE: Mini-Mental State Examination, PET: positron emission tomography.

### PET acquisition and image processing

The radioligand, ^18^F-FDG, was synthesized using a PET synthesizer (Sumitomo Heavy Industries, Ltd., Tokyo, Japan). The radiochemical purity of ^18^F-FDG was greater than 95%. The PET scanning was performed at the Tokyo Metropolitan Institute of Gerontology using a SET-2400W scanner (Shimadzu Corporation, Kyoto, Japan) in the three-dimensional mode. The in-plane and axial resolutions of the full width at half maximum were 4.4 mm and 6.5 mm, respectively. The transmission data were acquired with a rotating ^68^Ga/^68^Ge rod source for measured attenuation correction. The emission data were acquired for 6 min starting 45 min after an intravenous bolus injection of approximately 150 MBq of ^18^F-FDG. Sixty-three-slice images with a voxel size of 2.054 × 2.054 × 3.125 mm^3^ and matrix size of 128 × 128 were obtained. The data were reconstructed after correcting for decay, attenuation, and scatter.

**Figure 1:**
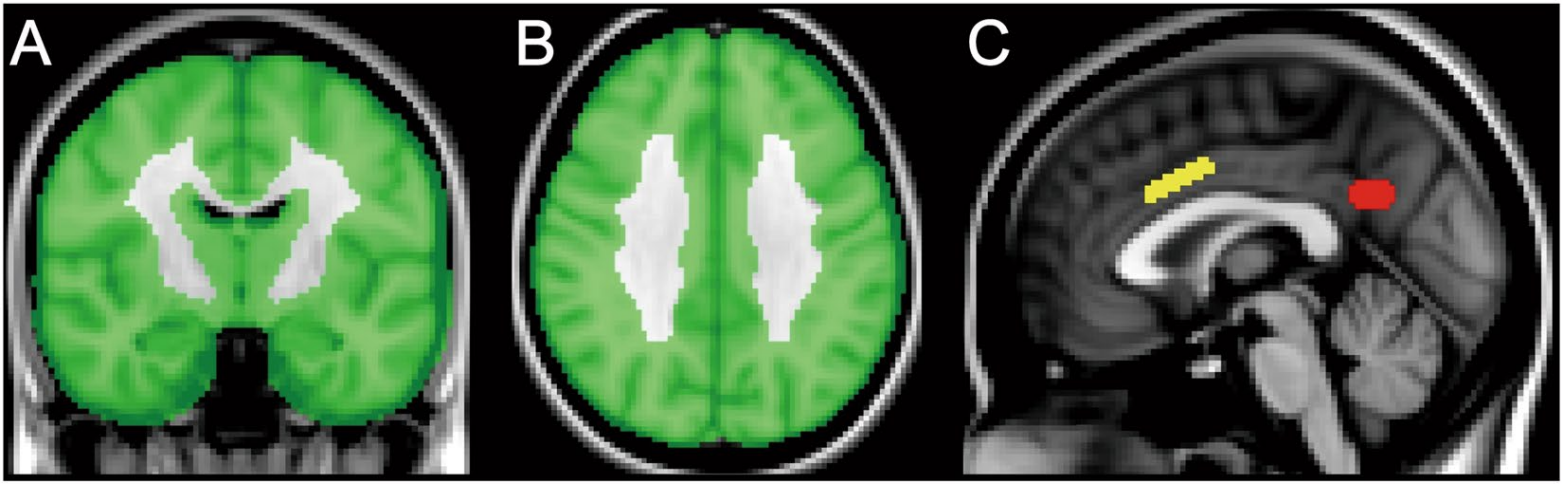
Mask and volumes-of-interest (VOIs) on the Montreal Neurological Institute standard brain. A cortical mask was created for the voxel-wise analysis, and is displayed in the coronal (**A**) and axial (**B**) sections. The VOIs placed on the anterior cingulate cortex (**C**: yellow) and posterior cingulate cortex/precuneus (**C**: red) are displayed in the sagittal section.

The images were processed using the FMRIB Software Library (version 5.0.4; Oxford University, Oxford, UK). All ^18^F-FDG images were nonlinearly transformed into the Montreal Neurological Institute (MNI) standard space from the native space using an in-house developed ^18^F-FDG template. The transformed images were globally normalized using a cortical mask developed in-house as a reference region (Fig. 1A,B). The mean value within the masked voxels was set to 1, and the normalized images were smoothed with an isotropic Gaussian kernel with a sigma of 4 mm for the subsequent voxel-wise and volume-of-interest (VOI) analyses.

### Longitudinal analysis

An exploratory whole-brain voxel-wise analysis was performed to assess the effects of aging on ^18^F-FDG distribution by comparing the baseline and second PET scans as well as the effects of the ApoE ε4 genotype on ^18^F-FDG distribution using Statistical Parametric Mapping (version 12; Wellcome Trust Center for Neuroscience, London, UK) and bspmview (http://www.bobspunt.com/bspmview), implemented in MATLAB (version R2014a; The MathWorks, Inc., Natick, MA. USA). A two-way repeated-measures analysis of variance with a full factorial design consisting of two time points (baseline and second PET) × two conditions (presence or absence of the ApoE ε4 genotype) was performed. We also specified sex and MMSE scores as nuisance covariates to exclude any of their effects. Correction for multiple comparisons was applied to the whole-brain voxel-wise analysis using a family-wise error (FWE) approach. The FWE-corrected threshold was set at *p* < 0.05.

A VOI analysis was then performed to test the longitudinal association between ^18^F-FDG uptake and age, and the influence of ApoE ε4 genotype on ^18^F-FDG distribution. First, VOIs were manually placed on the ACC and posterior cingulate cortex/precuneus (PCC/PC) in the MNI standard space, where voxel-wise analysis detected highly significant clusters (Fig. 1C). The VOI volumes were 992 mm^3^ (124 voxels) and 784 mm^3^ (98 voxels) for the ACC and PCC/PC, respectively. These VOIs were superimposed on normalized ^18^F-FDG images in the MNI standard space, and the values for the VOIs were extracted. For each of the ACC and PCC/PC VOIs, we also calculated the annual rate of reduction in normalized ^18^F-FDG uptake (%) as follows: 100 × [(VOI value at baseline) − (VOI value at second PET)]/(VOI value at baseline)/(time interval from baseline to second PET). The annual rates in the ACC and PCC/PC were compared between groups 2 and 3 (i.e., presence or absence of ApoE ε4 genotype) using Student’s *t*-tests. *P* values less than 0.05 were considered statistically significant.

### Cross-sectional analysis

In order to integrate the results of the longitudinal analysis, a cross-sectional analysis was performed on the data from the baseline and second PET scans and VOIs described above. We performed a correlational analysis of the relationship between normalized ^18^F-FDG uptake and age in the ACC and PCC/PC. *P* values less than 0.05 were considered statistically significant. All statistical analyses were conducted using SPSS Statistics version 22 (IBM, Armonk, NY).

### Data Availability

The datasets generated during and/or analyzed during the current study are available from the corresponding author on reasonable request.

## Results

**Figure 2:**
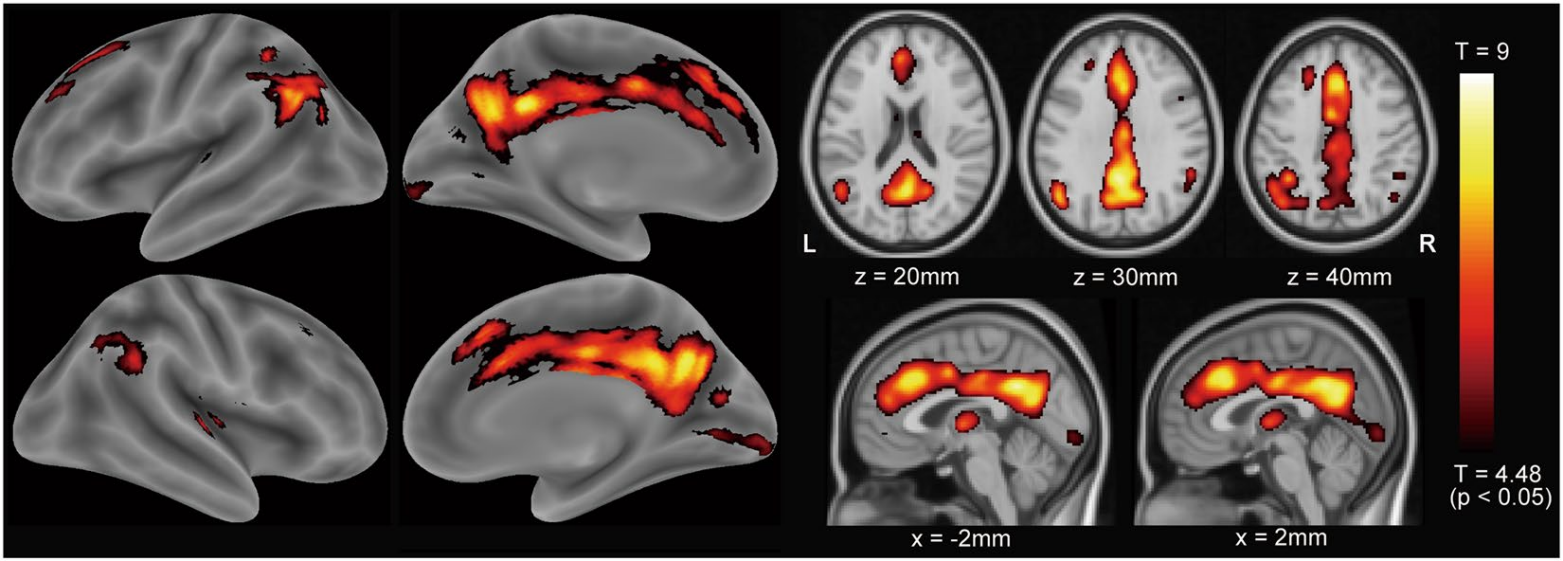
Results of the voxel-wise analysis: effects of aging on ^18^F-FDG distribution. To assess the effects of aging and apolipoprotein E ε4 genotype, a voxel-wise analysis was performed on all participants using a two-way repeated-measures analysis of variance with two time points (baseline or second positron emission tomography scan) × two conditions (presence or absence of apolipoprotein E ε4 genotype). Sex and MMSE scores were specified as nuisance covariates. The aging effects on ^18^F-FDG distribution were significant at *p* < 0.05, familywise error rate-corrected. The significant clusters extended to the anterior cingulate cortex, posterior cingulate cortex/precuneus, and lateral parietal cortex. The hot scale represents the magnitude of the *p* and *t* values. ^18^F-FDG: fluorine-18-labeled fluorodeoxyglucose, R: right, L: left, MMSE: Mini-Mental State Examination.

The exploratory whole-brain voxel-wise analysis using a two-way repeated-measures analysis of variance revealed significant effects of aging on ^18^F-FDG distribution at *p* < 0.05, FWE-corrected (Fig. 2). The clusters that extended through the ACC, PCC/PC, and lateral parietal cortex (LPC) were highly significant, suggesting that ^18^F-FDG uptake decreased in these areas with advancing age. The cluster size in the ACC and PCC/PC was 10737 voxels. The significant clusters in the left and right LPC were 2070 voxels and 568 voxels, respectively. However, the effects of the ApoE ε4 genotype on ^18^F-FDG distribution were not significant in both the positive and negative contrasts at *p* < 0.05, FWE-corrected.

**Figure 3:**
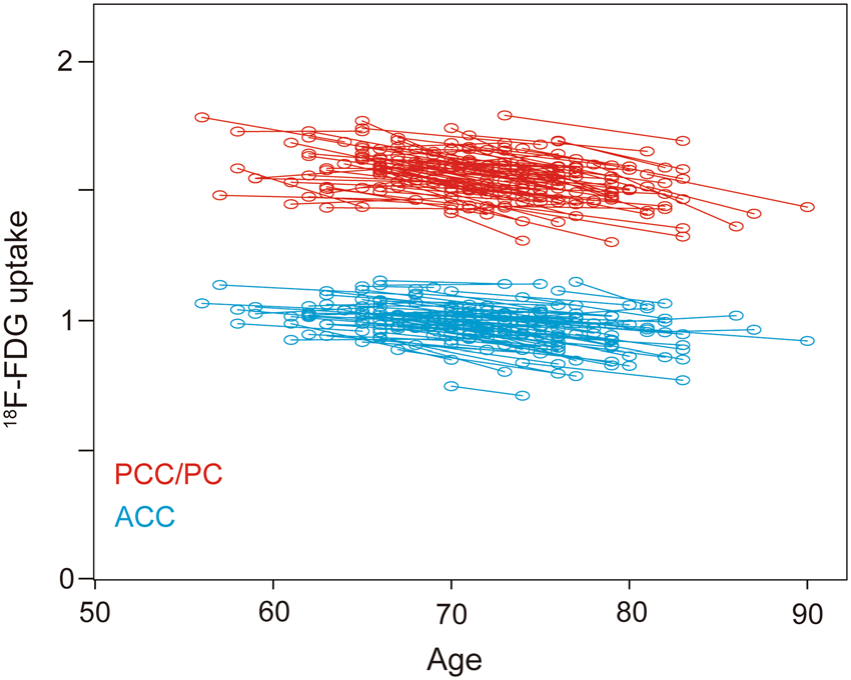
Scatter plots depicting longitudinal changes in ^18^F-FDG uptake (n = 107). The shift in ^18^F-FDG uptake from the baseline PET scan to the second PET scan is shown at the participant level by the slopes connecting paired circles. The blue and red circles represent ^18^F-FDG uptake in the ACC and PCC/PC, respectively. ^18^F-FDG: fluorine-18-labeled fluorodeoxyglucose, ACC: anterior cingulate cortex, PCC/PC: posterior cingulate cortex/precuneus, PET: positron emission tomography.

**Figure 4:**
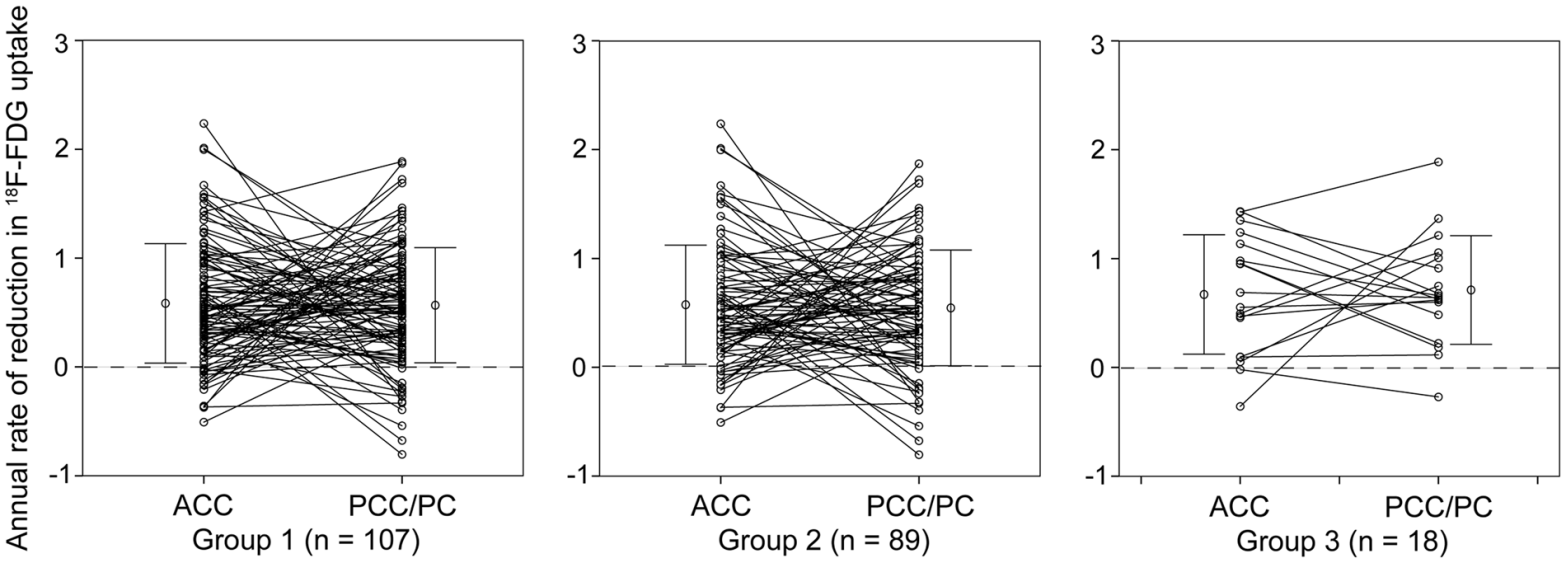
Annual rates of ^18^F-FDG reduction (%) in the ACC and PCC/PC. The slopes connecting paired circles between the ACC and PCC/PC demonstrate the annual rates of the ^18^F-FDG decreases in each participant. Group 1 included all participants (n = 107). Group 2 consisted of participants without the apolipoprotein E ε4 genotype (n = 89). Group 3 consisted of participants with the apolipoprotein E ε4 genotype (n = 18). The error bar represents mean ± SD. ^18^F-FDG: fluorine-18-labeled fluorodeoxyglucose, ACC: anterior cingulate cortex, PCC/PC: posterior cingulate cortex/precuneus.

The results of the longitudinal VOI analysis of the data from the ACC and PCC/PC are displayed in Figs 3 and 4. For each participant, the changes in ^18^F-FDG uptake from the baseline scan to the second PET scan are plotted in Fig. 3. We visually confirmed that these results were consistent with the results shown in Fig. 2, which suggested that ^18^F-FDG uptake in the ACC and PCC/PC tended to decrease with advancing age. The annual rates of reduction in ^18^F-FDG uptake were plotted in the ACC and PCC/PC for each group (Fig. 4). The rates in the AC and PCC/PC in the group-1 individuals were 0.58 ± 0.55% and 0.57 ± 0.53% (mean ± SD), respectively. The corresponding rates were 0.57 ± 0.55% and 0.54 ± 0.53%, respectively, in the group-2 individuals, and 0.67 ± 0.55% and 0.71 ± 0.50%, respectively, in the group-3 individuals. The annual rates did not significantly differ between the group-2 and -3 individuals in the ACC (*p* = 0.49) and PCC/PC (*p* = 0.20).

**Figure 5:**
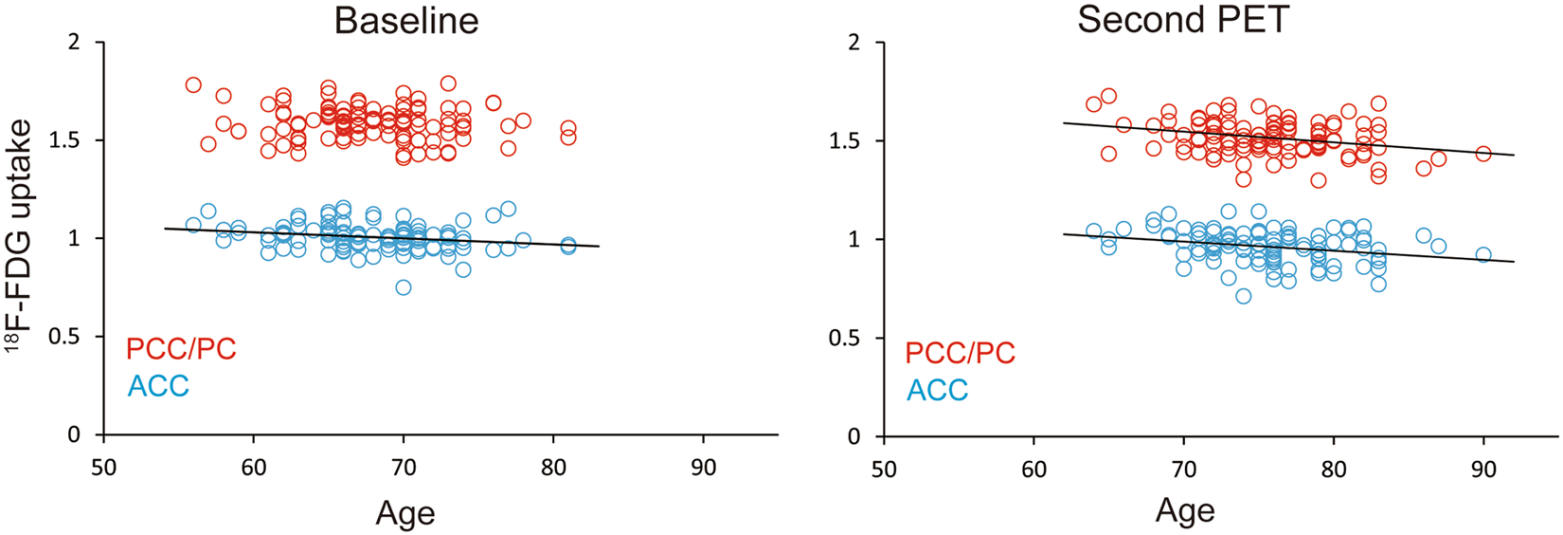
Scatter plots depicting the relationship between ^18^F-FDG uptake and age at the baseline and second PET scans in all participants (n = 108). The blue and red circles represent ^18^F-FDG uptake in the ACC and PCC/PC, respectively. The results of the baseline scan (left) show a significant correlation in the ACC (r = 0.233, *p* = 0.016) but not the PCC/PC (r = 0.114, *p* = 0.240). The second PET scan (right) revealed significant correlations in the ACC (r = 0.274, *p* = 0.004) and PCC/PC (r = 0.303, *p* = 0.002). A solid line represents a simple regression line between the two variables. ^18^F-FDG: fluorine-18-labeled fluorodeoxyglucose, ACC: anterior cingulate cortex, PCC/PC: posterior cingulate cortex/precuneus, PET: positron emission tomography.

The results of the cross-sectional analysis of the VOIs in the ACC and PCC/PC are displayed in Fig. 5. The relationships between ^18^F-FDG uptake and age at the baseline and second PET scans were plotted in the ACC and PCC/PC. In the baseline scans (Fig. 5, left), a significant correlation was found in the ACC (r = 0.233, *p* = 0.016) but not the PCC/PC (r = 0.114, *p* = 0.240). In contrast, significant correlations were found in the ACC (r = 0.274, *p* = 0.004) and PCC/PC (r = 0.303, *p* = 0.002) in the second PET scans (Fig. 5, right).

## Discussion

The primary objective of this study was to investigate the longitudinal effects of aging on ^18^F-FDG distribution in the brains of cognitively normal elderly individuals. We showed that ^18^F-FDG uptake in the ACC, PCC/PC, and LPC was longitudinally decreased with higher statistical significance in elderly individuals. The follow-up period ranged from 4 to 11 years with a mean of 7.8 years. To the best of our knowledge, this is the first longitudinal study investigating aging effects on ^18^F-FDG distribution in a large number of elderly individuals with a longer follow-up period. Recent studies addressing aging effects on ^18^F-FDG distribution^13–15^ have focused primarily on the progression of AD, with fewer participants and shorter follow-up periods. In a study by Shokouhi and colleagues with a mean follow up on 4.3 years, ^18^F-FDG distribution was fairly stable over time in 24 healthy elderly participants^15^. Similarly, no regional changes in ^18^F-FDG distribution were observed in a study by Ossenkoppele and colleagues with 11 healthy elderly participants and mean follow-up period of 2.5 years^14^. These may indicate that a follow-up period of 2–4 years is too short to investigate aging effects on ^18^F-FDG distribution in healthy elderly individuals. On the other hand, recent cross-sectional ^18^F-FDG PET studies in young to elderly individuals have consistently shown that the most prominent age-associated decrease in ^18^F-FDG uptake was in anterior regions of the brain, including the ACC, and that the LPC is one of the areas in which ^18^F-FDG uptake decreases with advancing aging^5–9^. Recent studies have also reported that part of the PCC/PC is an area that exhibits an age-associated decrease in ^18^F-FDG uptake, with relatively low statistical significance^6–8^. Therefore, our results were in line with those of previous cross-sectional ^18^F-FDG PET studies.

Compared to the results of the cross-sectional studies that investigated the association between ^18^F-FDG uptake and aging in young to elderly individuals, the present longitudinal study detected a highly significant association in the PCC/PC in addition to the ACC (Fig. 2). These findings may be explained as follows. The magnitude of the ^18^F-FDG decrease in younger to elderly individuals is much larger in the ACC than it is in the PCC/PC, while the magnitude of the ^18^F-FDG decrease in the ACC is comparable to the decrease in the PCC/PC over old age. In fact, the differences in glucose metabolism between young and elderly individuals tend to be greater in anterior regions of the brain^2^. Fjell and colleagues recently investigated the effects of aging on regional brain volume in a cross-sectional study of 1,100 healthy adults (18–94 years), and they identified 3 basic types of age-associated trajectories: (1) a linear reduction, (2) stability followed by a decline, and (3) a steep nonlinear decline^21^. They showed that the volumes in the amygdala, nucleus accumbens, and striatum decreased linearly in an age-associated manner from young adulthood. After a period of relative stability during middle age, the volume of the hippocampus continuously decreased over old age.

**Figure 6:**
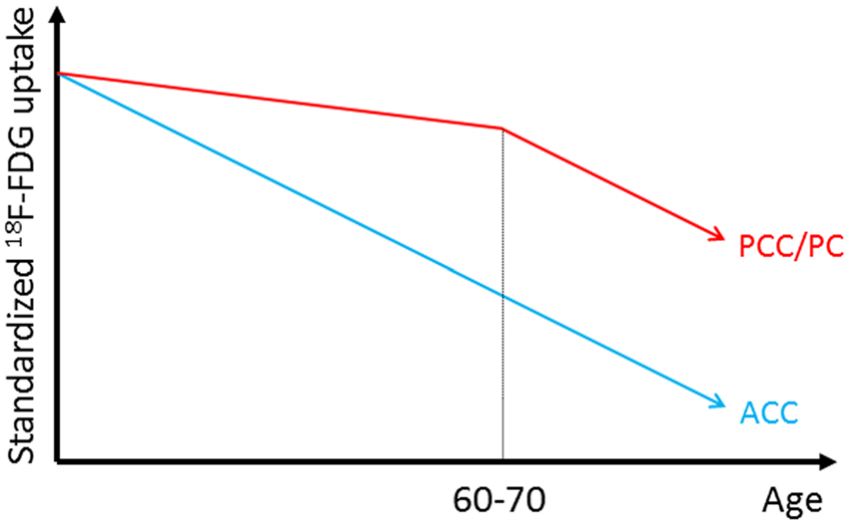
Schematic model of the age-associated trajectories. The blue and red lines represent the age-associated decreases in ^18^F-FDG uptake in the ACC and PCC/PC, respectively. In the ACC (blue), ^18^F-FDG uptake decreased continuously over time. In the PCC/PC, ^18^F-FDG uptake was relatively stable before age 60–70 and then started to decease in an age-associated manner. ^18^F-FDG: fluorine-18-labeled fluorodeoxyglucose, ACC: anterior cingulate cortex, PCC/PC: posterior cingulate cortex/precuneus.

Interestingly, as part of the temporoparietal networks, the PCC/PC is functionally connected with the hippocampus^22–24^, and, as part of the frontostriatal networks, the ACC is functionally connected with the ventral striatum, including the nucleus accumbens^23,25^. In these networks, changes in regional volume with advancing age^21^ may remotely affect regional metabolic changes. Thus, a linear volumetric reduction in the nucleus accumbens may cause linear metabolic reductions in the ACC, and stability followed by an accelerated volumetric decline in the hippocampus may similarly cause metabolic reductions in the PCC/PC. These speculations might be supported by the results shown in Fig. 5 that ^18^F-FDG uptake decreased continuously in the ACC, while ^18^F-FDG uptake in the PCC/PC was stable until around age 60–70 but then started to decease in an age-associated manner. Based on previous findings and our results, we propose a model of the effects of aging on ^18^F-FDG uptake in the ACC and PCC/PC (Fig. 6), although future studies are needed to validate this speculative model.

One of the limitations of the present study was the absence of amyloid-β (Aβ) PET imaging in the cognitively normal elderly participants. Because the PCC/PC and LPC are areas that exhibit decreased ^18^F-FDG uptake in patients with AD^26,27^, one cannot deny that some of the participants in this study might have already been in a preclinical stage of AD. Previous Aβ PET imaging studies have found that about 10–30% of cognitively normal elderly participants present significant Aβ deposition^28–32^. The ApoE ε4 genotype is associated with higher Aβ retention^29^, and possibly causes AD-like hypometabolism in cognitively normal elderly participants^33^. Therefore, in order to exclude as many participants as possible who might be in a preclinical stage of AD, we set up group 2, which consisted of participants without the ApoE ε4 genotype. This allowed us to assess the influence of ApoE ε4 genotype on ^18^F-FDG distribution. The results of the voxel-wise analysis and comparison of the annual rates of reduction in ^18^F-FDG uptake (Fig. 4) suggest that the longitudinal ^18^F-FDG reduction in the ACC and PCC/PC occurs regardless of ApoE genotype. However, because our sample size was unequal between groups 2 and 3, further investigations are needed to validate our results.

Aβ deposition reportedly starts to develop more than 20 years before the onset of cognitive decline, which suggests that the preclinical stage of AD can last over than 20 years^13,32,34^. Additionally, ^18^F-FDG uptake in the PC, which shows the earliest metabolic decline in the brain, reportedly starts to decrease about 14 years before AD onset^13^. Some longitudinal studies have found that cognitively normal individuals with Aβ deposition are at higher risk for future cognitive decline than those without Aβ deposition^35,36^. However, such a long duration of the preclinical stage of AD suggests that the preclinical stage of AD might be part of the normal aging process in some elderly individuals^23^. The differences between normally aging individuals and cognitively normal elderly individuals with Aβ deposition or other latent diseases are still unclear, which makes it difficult to define normal aging. In other words, identifying elderly individuals who might be in a preclinical stage of undetected dementia is extremely difficult. Additional longitudinal follow-up investigations of the participants in this study will be needed to further elucidate this issue.

The PCC/PC and LPC are the main components of the default mode network (DMN), which plays an important role in regulating complex cognition and behavior^37–39^. Its function is affected by aging^23,40^. Thus, the DMN is vulnerable to aging, and functional disruption in the DMN has been reported to be associated with cognitive decline in aging^41^. Considering the relationship between the DMN and aging, our findings of the longitudinal decreases in ^18^F-FDG in the PCC/PC and LPC might partially reflect aging-induced reductions in the functional connectivity of the DMN.

Another limitation of this study was that we could not perform the MRI-based partial volume correction (PVC) due to the absence of adequate MRI data. Because the mean interval between PET scans was 7.8 years, the volume of the brain likely decreased over the interval of this study. Therefore, the lack of PVC method might affect ^18^F-FDG measurements due to aging related atrophy. This limitation needs to be resolved in subsequent studies.

Both males and females show age-associated ^18^F-FDG decline, predominantly in anterior regions of the brain^6,8,12^. However, sex differences in aging effects on ^18^F-FDG distribution are controversial. Some researchers suggest that sex differences are non-significant or minimal; however, others suggest that males show age-associated ^18^F-FDG decline in wider cortical areas than females^5,12^. Furthermore, sex differences in ^18^F-FDG uptake may depend on age; the difference may be smaller in elderly compared to middle aged people^6^. We could not address sex differences in this study because the number of people based on sex in our sample was unequal. Further investigations are also needed to address this issue.

## Conclusions

The results of the present longitudinal study showed that ^18^F-FDG uptake in the ACC, PCC/PC, and LPC of cognitively normal elderly individuals decreased highly significantly with advancing age and that these findings remained regardless of ApoE ε4 genotype. The cross-sectional results showed that ^18^F-FDG uptake in the ACC decreased continuously with age, while ^18^F-FDG uptake in the PCC/PC was stable until around age 60–70 at which point it began to decease. These findings suggest that the trajectories of age-associated decreases in ^18^F-FDG uptake differ between the ACC and PCC/PC. However, because of the limitations of this study, such as the absence of Aβ assessments, additional longitudinal follow-up investigations in this sample are needed to confirm that our findings are due to aging effects.
